# Exploiting nanoscale effects in phase change memories

**DOI:** 10.1039/c8fd00119g

**Published:** 2018-11-07

**Authors:** Benedikt Kersting, Martin Salinga

**Affiliations:** a Institute of Physics (IA) , Physics of New Materials , RWTH Aachen University , Sommerfeldstr. 14 , Aachen 52074 , Germany . Email: martin.salinga@rwth-aachen.de

## Abstract

The market launch of Intel’s 3D XPoint™ proves phase change technology has grown mature. Besides storing information in a fast and non-volatile way, phase change memories (PCMs) may facilitate neuromorphic and in-memory computing. In order to establish PCM as a lasting element of the electronics ecosystem, scalability to future technology nodes needs to be assured. Continued miniaturization of PCM devices is not only prescribed in order to achieve memories with higher data density and neuromorphic hardware capable of processing larger amounts of information. Smaller PCM elements are also incentivized by the prospect of increased power efficiency per operation as less material needs to be heated up for switching. For this reason, a good understanding of the effects of confinement on phase change materials is crucial. Here we describe how miniaturization increases the importance of interface effects and we show how in consequence the crystallization kinetics of phase change materials, when confined into nanometer sized structures, can change significantly. Based on this analysis, the implications of such nanoscale effects are discussed and possible ways of exploiting them proposed.

## Abstract 2

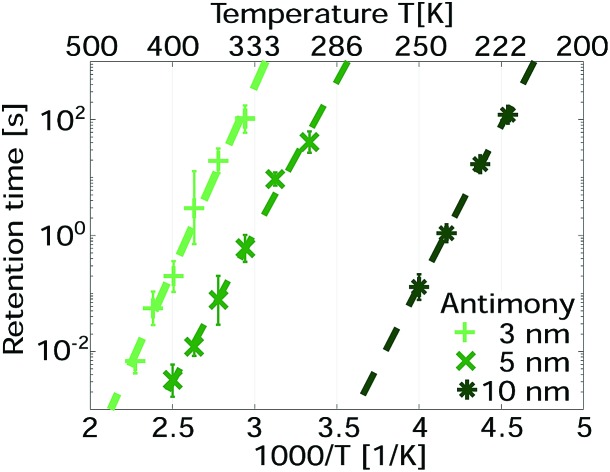
Nano-confined phase change memory cells based on pure Sb have been electrically characterized.

## Introduction

Switching in phase change memories is an electrically initiated but ultimately thermally driven process in both directions: amorphization *via* melt-quenching and crystallization through annealing. The progress towards nanoscale cell dimensions is not only following requirements regarding device density, in the case of PCM it is also incentivized by the promise to achieve chips with lower power consumption.^1^-^4^ Smaller volumes of phase change material have a smaller heat capacity, *i.e.* need less energy to be molten or annealed to a given temperature. Also, a smaller volume can be fully crystallized in less time without the need to increase the crystal growth velocity. As a consequence, electrical pulses may be less intense and shorter, both reducing the required energy per switching event. Fully confined cell structures^3^ do not only allow the shrinking of in particular the cross-sectional area of the electrodes so that the absolute currents required for switching the cell can be reduced: their thermal interfaces with dielectric materials also keep the Joule heat concentrated within the phase change material. Hence, the increase of the interface-to-volume ratio of a phase change memory cell has an immediate impact on its thermal properties and its electrical demands.

Attempts to leap forward in scaling the contact cross-section to the nanometer range, *e.g.* by using carbon nanotubes as electrodes, imply that the effects of scaling may indeed continue along those trends that have been observed in several device generations before.^5^-^6^ However, when the actual volume of phase change material is confined more and more narrowly, even properties supposedly characteristic for a material begin to change. Most noticeably, both structural phase transitions, melting and crystallizing, have been reported to take place at significantly different temperatures than in bulk (for this purpose that means in 100 nm thin films).^4^,^7^-^9^

Often in the past, size effects were observed without having sealed the nanostructures with cladding. Noé *et al.*^10^ recently demonstrated how easily exposure to an oxygen atmosphere can lead to crucial oxidation of the surface of typical phase change materials and drastically change the crystallization behavior as a consequence of the compositional change. Conversely, careful *in situ* capping, even with silicon-oxide, can keep the phase change material’s composition unaltered.^10^ When introducing a certain cladding, however, the material choice itself turns out to have an influence on the crystallization properties.

Traditionally, the interfacial energies between the cladding and the different phases of the phase change material have been seen as the origin for heterogeneous nucleation at the interface, which can largely dominate over homogeneous nucleation within the disordered phase change material, especially when the interface-to-volume ratio is large. Even crystal growth along such an interface can be sped up or slowed down compared to its bulk values depending on the interfacial energies.^11^

In general, crystal growth becomes more dominant in the crystallization process of nanometric phase change memory cells, not only compared to homogeneous, but even to heterogeneous nucleation. As soon as a crystal-to-glass interface exists only a few nanometers away from any group of disordered atoms, they are much more likely to be incorporated into that growing crystal than they are able to reorganize themselves into a new crystal nucleus first.

Besides reduced nucleation probability in a smaller volume and the altered heterogeneous nucleation at the interface, the confinement of a material can have an impact on the ease with which the atoms can reconfigure within a nanometric volume. Researchers at Yale nicely discussed these competing effects when studying crystallization of metallic glass nanorods of different sizes.^12^–^14^ The apparent viscosity is reduced as the confinement is narrowed. As a consequence, crystallization kinetics are slowed down in smaller structures. In the field of phase change materials, this idea has also been proposed to interpret the effects of confinement.^11^

Others (*e.g.*ref. 15) put crystallization-induced stress into the center of their interpretation of what happens upon confinement, acknowledging the significant difference in mass density between disordered and crystalline phases. Due to the inability of atoms to freely rearrange along a surface in the presence of a capping, they argue that the necessary viscous flow is more strongly coupled with crystal growth.

A different argument is made by Simpson *et al.*^16^ when interpreting their observation of increased crystallization temperature in thinner films confined by an encapsulation material that exerts compressive stress on the initially amorphous phase change material. Based on the observation of vacancies in crystalline phase change materials, they believe that under too high a compressive stress the material might not have sufficient space to realize such a state. In contrast, Eising *et al.*^17^ have demonstrated convincingly how compressive stress accelerates crystal growth, which seems intuitive in view of the higher mass density of the crystalline state.

While stress certainly does have an influence on the crystallization kinetics, it is less clear how important this is compared to other effects like the increased viscosity due to confinement or parameters controlling it. In a series of molecular-dynamics simulations Scheidler *et al.*^18^,^19^ investigated cooperative motion in supercooled liquids in close proximity of confining walls. The roughness or smoothness of these surfaces turned out to play a decisive role, pointing to a physical quantity that might turn out to be a relevant specifier for comparing different claddings in the future.

Several of the above-mentioned aspects must be expected to depend (quantitatively) on how the amorphous phase change material and its confinement was created: by deposition from the gas phase and subsequent addition of cladding material or by quenching the melt within its confinement.^20^ The literature quantifying the crystallization kinetics of phase change materials for melt-quenched amorphous materials is very limited. When it comes to confinement effects, there is an even stronger need for research on the technologically relevant, melt-quenched state.

While subject to the exact choice of cladding material, the general trend in phase change materials is an increased stability of the disordered states, *i.e.* slowed-down crystallization kinetics, upon confinement. If one does not want to end up with switching speeds and retention times orders of magnitude too high for useful applications, one may not continue to use the same materials that have been designed to work in significantly larger memory cells. Instead, when scaling towards the size of very few nanometers one should consider materials which crystallize much too quickly in larger volumes. They might even be difficult to be amorphized in bulk. Pure antimony fulfills this criterion. In the following we will describe and analyze experiments in which nano-confined phase change memory cells based on pure Sb have been electrically characterized.

## Experimental methods

### Device geometry

**Fig. 1:**
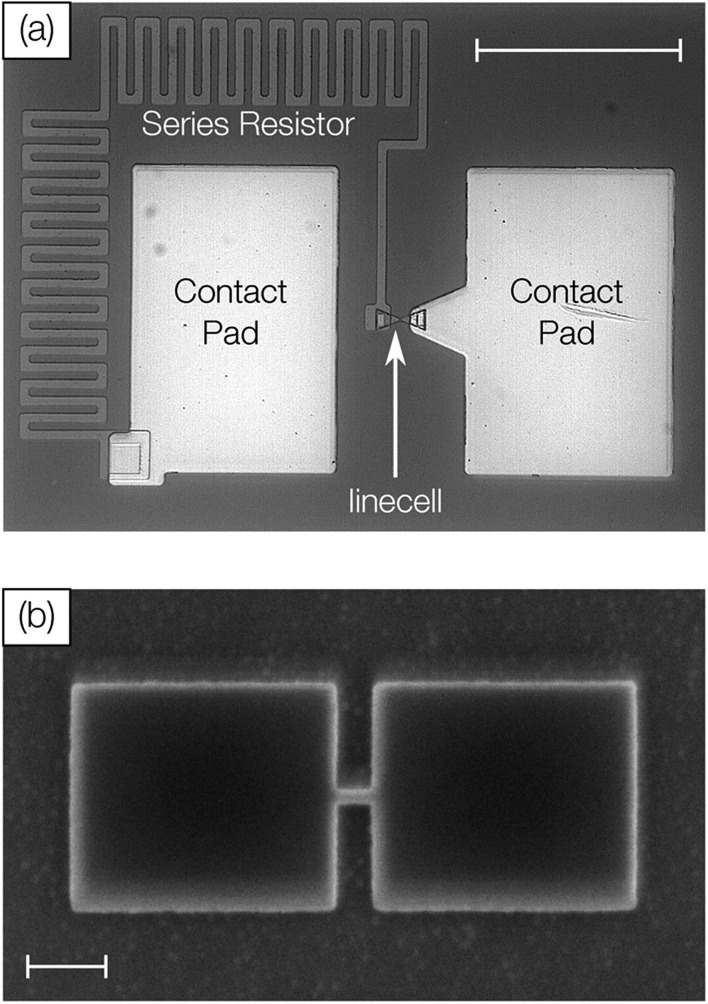
Optical microscopy image of the complete device structure (a) and SEM image of the linecell geometry (b). The SEM image shows the developed HSQ-resist that is used to pattern the capped phase change material. After patterning the structure is immediately covered with an additional 18 nm of sputtered SiO_2_. Thus, the device geometry is controlled before that fabrication step. The scale bar in (a) is 60 μm long and the scale bar in (b) 200 nm.

Device structures were fabricated on a silicon substrate with 40 nm thermally grown SiO_2_. The device comprises gold contact pads, a titanium (series) resistor and a linecell of pure antimony (Fig. 1a). Atom-probe tomography confirmed the purity of sputter-deposited thin films (Sb > 99.9%). In three different samples the antimony is confined to 10 nm, 5 nm and 3 nm. The center of the linecell is 50 nm wide and 100 nm long (Fig. 1b). Upon electrical excitation Joule heating will increase the temperature of this active volume above the melting temperature. The on-chip series resistor (2–4 kΩ) is supposed to reduce the device current when the linecell switches from high (MΩ) to low (kΩ) resistive states. Gold contact pads assure a reliable electrical contact at low ambient temperatures (100 K).^21^

### Probe system

The experiments were performed in a cryogenic probe station (JANIS ST-500-2-UHT). In this system the ambient temperature is adjusted by cooling with liquid nitrogen and two resistance heaters. The temperature is probed with Lakeshore Si DT-670B-CU-HT diodes (accuracy < 0.5 K) at four positions within the chamber and controlled with a Lakeshore 336 Automatic Temperature Controller. To avoid water condensation and heat transfer *via* convection the probe chamber is evacuated to 10^–5^ mbar. Cooling braids from a copper chuck holding the sample are connected to a high-frequency Cascade Microtech Dual-Z probe, which is used to contact our devices, preventing heating of the sample by the probes.

### Electrical excitation

**Fig. 2:**
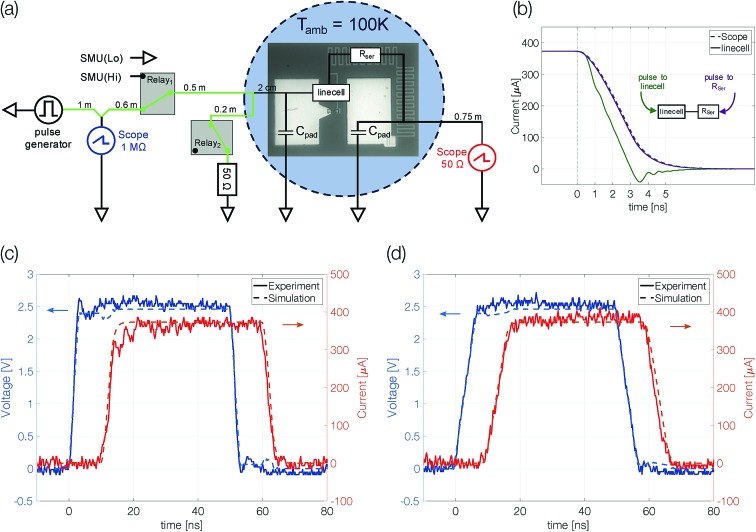
(a) Sketch of the electrical circuit in AC configuration. The 50 Ω terminated transmission path is marked green. In DC configuration both relays are switched; Relay two is open. (b) The simulation reveals that the transient signal passing through the linecell depends on which side the pulse is applied from (full lines). This difference cannot be detected in the scope (dashed lines) because the signal arriving at the scope always passes the RC-filter. The simulation of the circuit is capable of reproducing the experimental results for 50 ns wide pulses with 3 ns (c) and 7 ns (d) leading/trailing edges. Voltage (blue) and current (red) signals are shifted in the plot by 10 ns for better data visibility.

The electrical characterization of antimony linecell structures requires a combination of high current resolution (nA) and precise control of fast transient current signals (ns timescale). To achieve both requirements we combine a DC measurement path (Keithley 2400 Source Measure Unit) for the high current resolution measurements (*V*_read_ = 0.1 V) with an AC measurement path for the transient signals (Fig. 2a). Voltage signals applied with an Agilent 81150A Pulse Function Arbitrary Generator are picked up before the sample at a 1 MΩ terminated oscilloscope channel (Tektronix TDS3054D & DPO5104). To avoid distortions of the voltage pulse a 50 Ω transmission line (marked green in Fig. 2a) is passed along the oscilloscope (with an impedance of 1 MΩ) and the sample (with an impedance of at least several kΩ) before it is terminated with a 50 Ω resistor. Due to the short distances between the transmission line and the impedance mismatch (2 cm) multiple reflections superimpose on the timescale of 100 ps. For this reason, signals with a transition time of 3 ns (the limit of the pulse generator) and above are practically undistorted. The device current is measured over another 50 Ω terminated scope channel. The desired measurement path is selected with mechanical relays (OMRON G6Z-1F-A).

In particular, a precise control of transient signals turned out to be crucial (see the section “Limits of programmability” and Fig. 4a). To gain deeper insight into the transient current passing the linecell we performed circuit simulations including setup and sample with Simulink (Simscape). The capacity of the contact pads (60 × 90 mm^2^, *d*_SiO_2__ 138 nm, *ε*_r_ = 3.9) is estimated to be around 1.42 pF. The series resistor, which is included in order to limit the current passing through the phase change device after electrical breakdown (*e.g.* upon threshold switching), is a combination of resistor (2300 Ω) and capacitor (∼2378 mm^2^; *d*_SiO_2__ = 58 nm; *ε*_r_ = 3.9; 1.35 pF). It is represented as a 1.2 mm long transmission line (*C* = 1.125 pF mm^–1^; *R* = 1917 Ω mm^–1^). The phase change material in the crystalline state is assumed to behave like a purely resistive element. Coaxial transmission lines leading to the probe-head in contact with the device are simulated with a capacity of 90 pF m^–1^.

There is a reasonable match between simulated and measured transient signals (Fig. 2c and d). The shape of the 50 ns pulses with edges as short as 3 ns is well preserved. The simulation allows not only the reproduction of the experimental data but also the study the transient signal passing the linecell. It turns out that the trailing edge of the pulse arriving at the linecell depends on which side the signal is applied from (Fig. 2b). If the signal is applied to the series resistor side of the sample, effectively an RC-filter is placed in front of the linecell. Thus, the voltage drop across the phase change material changes faster if the electrical signal is applied to the linecell and the series resistor is behind it.†
†The trailing edge of the voltage pulses applied to the linecell defines the electrical quench rate. Devices with 10 nm Sb could only be quenched if pulses with the fastest electrical trailing edges (3 ns) were applied from the linecell side. We performed all experiments in this configuration.

## Results & discussion

### Evidence for successful amorphization

Single-elemental, semi-metallic antimony is prone to crystallization.^22^,^23^ Consequently, the first question is: can amorphous antimony be created by quenching from the melt when confined in a nanoscale device structure? To answer this question, we performed preliminary experiments at an ambient temperature of 100 K. At reduced temperatures, recrystallization is slowed down, increasing the chance of observing an amorphous mark fully blocking the electrical current path in a sample.

**Fig. 3:**
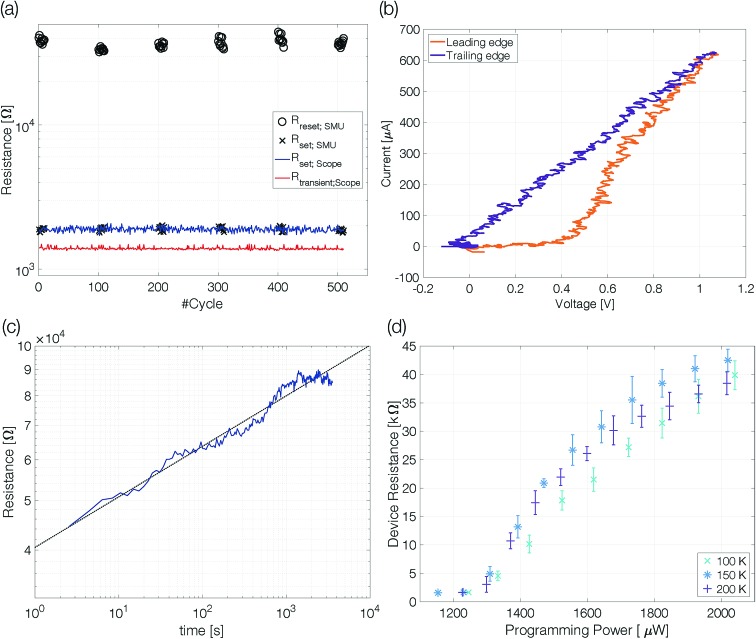
Amorphization of devices with 10 nm Sb. (a) Devices can be cycled reliably with an ON/OFF ratio of around 20. The set resistance (blue) and transient resistance (red) are calculated from transient signals. Every 100 cycles of the set and reset states are measured 10 times with an SMU. (b) Set pulse current–voltage characteristic of a 46 kΩ reset state. The device crystallizes during the pulse leading edge. (c) Resistance drift of a reset state (drive coefficient *ν* = 0.1 ± 0.02). The ambient temperature in (a), (b) and (c) was 100 K. (d) Programming curves at different ambient temperatures. The programming power to reset the device is increased step-wise from values too low to reset the device to a maximum value up to which the devices were found to operate reproducibly and reliably. This procedure is repeated five times. Error bars denote the standard deviation.

Samples with 10 nm Sb are reset to a high resistive state with trapezoidal voltage pulses (width 50 ns, edges 3 ns). During the plateau of the pulse the device resistance (*R*_transient;Scope_) is lower than the set resistance (Fig. 3a). The same is true for the resistivity of molten antimony [∼114 μΩ cm]^24^,^25^ compared to that of crystalline thin films [*r*_thinfilm_(*d* < 50 nm) > 150 μΩ cm].^26^ At low temperatures (100–225 K) the resistance of antimony linecells in the crystalline state was found to be almost temperature independent.^21^ The resistance decrease from *R*_set_ = 1850 Ω to *R*_transient_ = 1375 Ω gives a first hint of the applied power being sufficient to melt the phase change material. After the reset pulse the device ends up in a high resistive state (∼35 kΩ). Triangular pulses (200 ns leading and trailing edges) are used to switch the linecell back to the low resistive state. The device can be switched reliably with an ON/OFF ratio of around 20. A significantly increased resistance compared to that of the crystalline state is a first characteristic feature of an amorphized device.

The set process from the high resistive (amorphous) state to the thermodynamically stable crystalline state within a few nanoseconds requires substantial Joule heating to temperatures above the glass transition temperature. It is possible because the amorphous material switches to a low resistive state (on-state) at a threshold voltage.^27^ Threshold switching is of fundamental importance to operate phase change materials in an electronic memory. The current–voltage characteristics recorded during the set pulse suggest threshold switching between 0.4 V and 0.5 V, indicated by a pronounced kink in the current–voltage curve (Fig. 3b). The following rise of the device current is the result of growing Joule heating after threshold switching under a still increasing applied voltage. However, a gradual transition from high to low resistive states could also appear if a thin crystalline filament remained in the largely amorphized linecell. Hence, the 10 nm thick Sb possibly cannot be quenched fast enough to create an amorphous mark that blocks the complete cross-section of the linecell.

Resistance drift is another distinct characteristic of amorphous phase change materials. The resistivity increases with time as the melt-quenched material relaxes into energetically more favorable glassy states.^28^,^29^ The temporal evolution of resistance drift at constant temperature is commonly described by the equation *R*(*t*) = *R*(*t*_0_) × (*t*/*t*_0_)^*ν*^, with a constant drift exponent *ν*. A drift measurement over 3500 s shows a continuous resistance increase (Fig. 3c). At 100 K ambient temperature the size of the amorphous mark is stable and does not show indications of crystallization. The drift coefficient of 0.1 ± 0.02 is remarkably similar to that observed for other phase change materials at room temperature and above.^30^

Programming curves [*R*_Reset_(*P*_Programming_)] give insight into the power required to change the device state (Fig. 3d). The power applied to the device needs to be sufficient to melt the phase change material in a volume large enough to block the electrical current path through the device even after partial recrystallization in the course of melt-quenching. Above this programming onset the size of the amorphous mark in the device increases continuously with rising programming power; visible as increasing reset resistance.^21^

Programming onset (1275 ± 40 μW) and reset resistances above onset at different ambient temperatures (100 K, 150 K and 200 K) are comparable within the margin of error. Up to 200 K we detect an amorphous mark in the device. With changing ambient temperature the size of the molten volume (for the same power),^31^ crystal growth velocity,^20^,^31^ resistivity^21^ and drift coefficient^32^,^33^ may alter significantly. For this reason, we must refrain from drawing conclusions about the relative size of the amorphous mark obtained at different temperatures.

At 250 K no resistance change could be identified, which does not necessarily mean that it is no longer possible to quench a glass from the melt. Instead, the amorphous material might recrystallize within the 1.5 s it takes to switch from the AC-path (Reset) to the DC-path (Read).

### Device characteristics upon confinement

After confirming our general ability to amorphize Sb by quenching from the melt, we can now study how the device properties can be modified by confinement. Both the constraints on melt-quenching antimony (limits of programmability) and the stability against recrystallization (retention time) change significantly.

### Limits of programmability

At an ambient temperature of 100 K melt-quenched amorphous antimony is stable in the long-term against recrystallization. The programmability of a device is only limited by the power necessary to melt the phase change material and the quench rate required to avoid complete recrystallization. Both parameters can be controlled by the voltage pulses applied to the sample. The programming power (pulse amplitude) determines the size of the molten volume. The trailing edge of the reset pulse defines how abruptly the Joule heating in the device ends. The effective quench rate will depend also on how fast the heat is dissipated in the substrate, which is limited by the dielectric heat barrier out of SiO_2_.

**Fig. 4:**
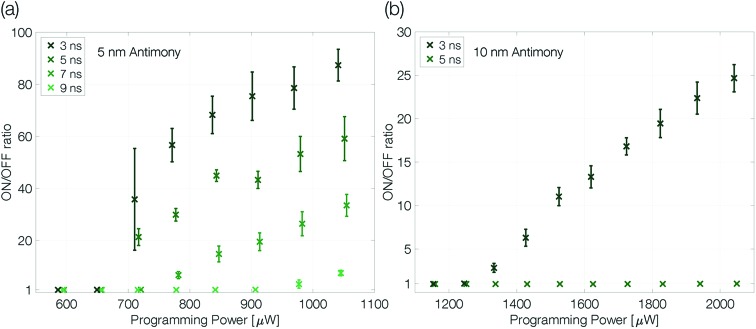
Programming curves of a 5 nm (a) and 10 nm (b) antimony device at 100 K ambient temperature. The ON/OFF ratio is given by *R*_Reset_/*R*_set_. The programming curves are measured as described previously (see the caption of Fig. 3). The trailing edge of the programming curves is increased in two nanosecond steps until the quench rate is too slow to create a change in linecell resistance; the pulse width is kept constant at 50 ns. Finally, to ensure the device did not degrade during the experiment a programming loop with the fastest trailing edge is repeated and reproduces the first. Error bars denote the standard deviation.

Extensive programming experiments with various pulse trailing edges show improved programmability for device structures that are confined more narrowly (Fig. 4). A reduction of the antimony thickness from 10 nm to 5 nm decreases the programming onset by a factor of almost 2, from 1300 μW to 675 μW (3 ns trailing edge). Less material needs to be molten and accordingly it requires less power to program the more narrowly confined devices.^1^,^2^

At the same time, the maximum ON/OFF ratio increases by a factor of 4 (compared at a programming power of around 50% above the programming onset). While the resistivity of crystalline antimony increases with decreasing film thickness,^26^ we do not have to expect large changes in the resistivity of amorphous thin films with different thicknesses as the typical mean free path in amorphous semiconductors is of the order of interatomic distances.^34^–^36^ Hence, the increased ON/OFF ratio is attributed mainly to a larger amorphous mark in the 5 nm Sb sample.

In addition, the quench rate that is necessary to suppress complete recrystallization of the initially molten material may be slower for a more narrowly confined material. Reset pulses with a 5 ns trailing edge do not end abruptly enough to decrease the conductance of the 10 nm Sb device, whereas the 5 nm Sb device can still be programmed even with 7 ns trailing edges. Sb-rich phase change materials are fast-growth materials with already low nucleation rates in much larger volumes.^24^ A small device volume like ours reduces the probability for nucleation even further and the rim of the small molten volume reaches an interface with crystalline antimony. Thus, no nucleation event is necessary for recrystallization: it can proceed purely by growth, *i.e.* by progression of the existing crystal boundary into the previously molten volume.

These two observations in more narrowly confined devices, *i.e.* an increased ON/OFF ratio and amorphization with slower pulse trailing edges, can be attributed either to a slower effective cooling rate (heat remaining longer) in the thicker device or a reduced crystal growth velocity upon confinement. The following section will demonstrate that the second effect is crucial.

### Confinement stabilizes the reset state

**Fig. 5:**
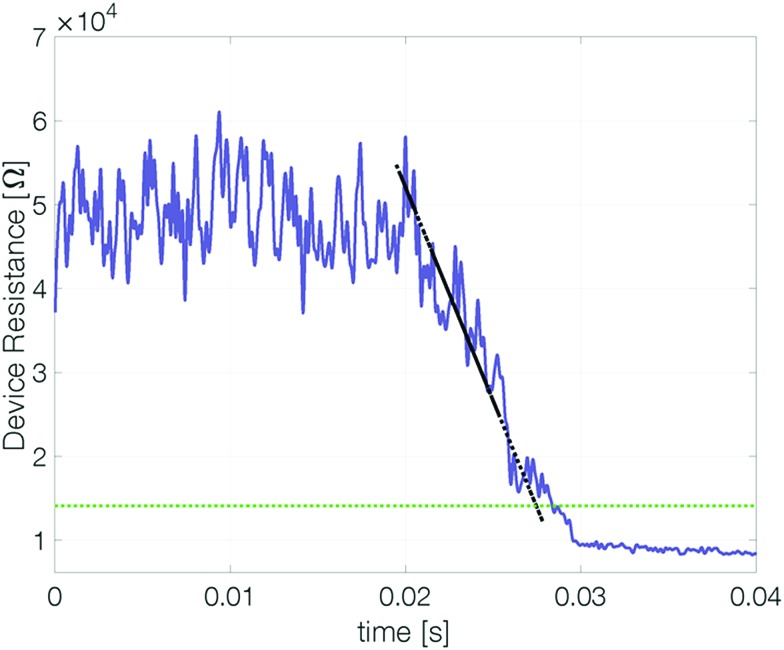
Evolution of the cell resistance after reset. Exemplary data of a 3 nm Sb device after reset at 420 K. The dashed green line marks the threshold resistance (2 × *R*_Cryst_) below which the reset state is no longer considered distinguishable from a set state. The retention time is defined as the intersection of the linearly fitted resistance decrease (dashed black line) with the threshold resistance level.

To determine whether a device based on pure antimony can be suitable for memory applications it is important to quantify how long (at least) two different states are distinguishable. To this end, our devices were reset and their resistance measured over time.‡
‡The cell resistance on timescales below 2 s was measured by applying a burst of 1000 triangular voltage pulses (with a peak voltage of 0.25 V). The current–voltage curve of each triangle is fitted linearly to obtain the device resistance. Measurements on longer timescales were realized with an SMU. The delay of the first SMU-based resistance read after reset is approximately 1.5 s. At elevated temperatures the reset resistance decreases continuously (approximately linearly) with time (Fig. 5). Recrystallization of the amorphous state limits the retention time.

To estimate the maximum retention time, we define the threshold resistance (2 × *R*_Cryst_) below which a reset state can no longer be reliably distinguished from the set state. A retention time merely obtained by a threshold value will, however, be sensitive to its exact value and discards all data except for a single point. By linearly fitting resistance over time the estimated retention time gets less sensitive to noise.§
§For the fit only data above the threshold resistance of twice the crystalline resistance (*R*_threshold_ = 2 × *R*_cryst_) are considered. If the resistance drops within the first 10 resistance measurements below the maximum measured resistance the data is fitted from the first measurement point. Otherwise the resistance is fitted from the point at which it drops for the first time below 0.9 times the maximum value. This procedure aims at fitting the experimental data only in a regime in which the resistance continuously decreases. Leaving out regimes where *e.g.* resistivity drift dominates the devices resistance avoids overestimating the retention time.


**Fig. 6:**
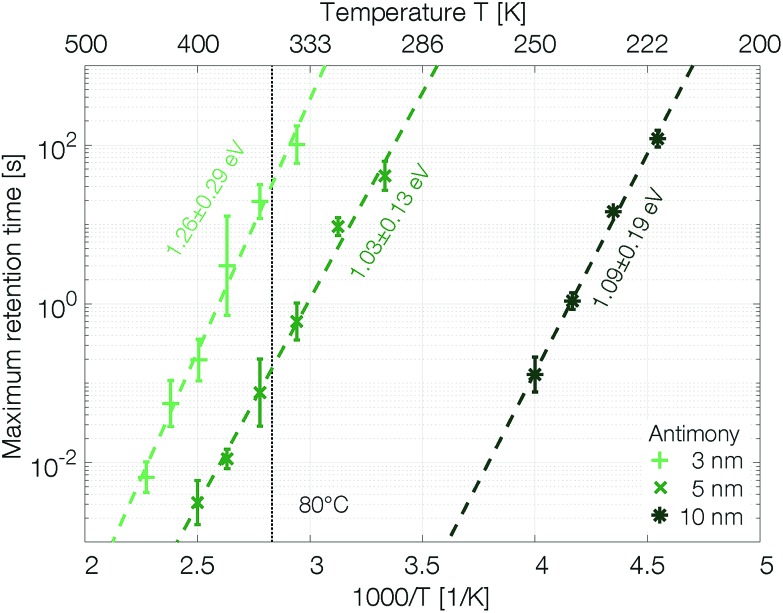
Confinement improves the retention time of single elemental antimony phase change devices by several orders of magnitude. To estimate the scattering of the retention time devices were melt-quenched at least five times at each temperature. The error is calculated on a log10-scale.

The retention time shows an Arrhenius temperature dependence over at least four orders of magnitude in time (Fig. 6). Following again the hypothesis of recrystallization taking place only from the rim towards the center of the amorphous mark, without the necessity of any nucleation event, the retention time is dominated by the crystal growth velocity. Samples with 10 nm Sb have an activation energy of 1.09 ± 0.19 eV. Confining the antimony from 10 nm to 5 nm and 3 nm increases the retention times by several orders of magnitude. The thickness reduction to 5 nm shifts comparable retention times to almost 100 K higher temperatures. Even stronger confinement to 3 nm boosts the retention time by another two orders of magnitude. This difference can certainly not be explained merely by a change of the size of the amorphous mark created in devices with different thicknesses of Sb. Instead, it demonstrates that the crystal growth velocity is significantly reduced in more narrowly confined material. The amorphous phase gets stabilized against crystallization.

The retention time of a sample confined to 3 nm is on the order of 32 s at 80 °C, which is 500 times the 64 ms DRAM refresh interval.^37^ Memory-mapped storage class memories (SCMs) start to become advantageous with retention times that are only ten times longer than that DRAM refresh interval.^38^ Strongly confined phase change memories of pure antimony are able to satisfy this specification.

## Conclusions

Extreme confinement increases the stability of the amorphous state against crystallization. Bulk material properties are outweighed by interfacial and confinement effects. Interfacial energy and mechanical stress might change the driving force for crystallization. Moreover, atomic mobility is restricted near the confining interfaces. An improved understanding of how different neighboring materials change the stability against crystallization or which of the mentioned effects are dominating will prove vital for future generations of phase change memory. Therefore, deeper insight into nanoscale confinement effects becomes highly relevant.

The attempt to pursue Moore’s Law will inevitably push device dimensions to scales where bulk properties no longer apply. In this regime materials previously not considered suitable for phase change memory applications can also become valid candidates for device applications. Instead of optimizing material compositions, interface-engineering may open new routes to enhance device performance.

## Conflicts of interest

There are no conflicts to declare.
